# A new species of Homoneura (Euhomoneura) from northern China (Diptera, Lauxaniidae)

**DOI:** 10.3897/zookeys.725.21267

**Published:** 2017-12-29

**Authors:** Li Shi, Xuefeng Gao, Wenliang Li

**Affiliations:** 1 College of Agronomy, Inner Mongolia Agricultural University, Hohhot 010019, China; 2 College of Forestry, Henan University of Science and Technology, Luoyang 471003, China

**Keywords:** *
Euhomoneura*, *Homoneura*, new record, new species, Palaearctic region

## Abstract

Homoneura (Euhomoneura) yanqingensis **sp. n.** is described as new to science and Homoneura (E.) shatalkini Papp, 1984 is recorded from China for the first time. Photographs and illustrations are provided for both of these species, including genitalia. A key is provided to separate the Chinese species of the subgenus Euhomoneura.

## Introduction

The subgenus Euhomoneura Malloch, 1927 is a small subgenus of the genus Homoneura (Wulp, 1891), which can be separated from other subgenera by the following key features: the lower margin of the face being about three times as wide as height of the gena and the anteriormost dorsocentral seta being at or before the transverse suture of the mesonotum. It includes 12 described species with three in China (Shiet al. 2012).

One species Homoneura (Euhomoneura) yanqingensis sp. n. is described as new to science and one species Homoneura (E.) shatalkini Papp, 1984 is newly recorded in China. A key is provided to separate the Chinese species of the subgenera Euhomoneura.

## Materials and methods

General terminology follows Gaimari and Silva (2010) and Shi and Yang (2014). Genitalia preparations were made by removing and macerating the apical portion of the abdomen in cold saturated NaOH for one hour, then rinsing and neutralizing them for dissection and study. After examination in glycerin, genitalia were transferred to fresh glycerine and stored in a microvial pinned below each specimen. Specimens examined were deposited in two collections: entomological collections of China Agricultural University, Beijing (**CAUC**) and Inner Mongolia Agricultural University, Hohhot (**IMAU**).

## Taxonomy

### Homoneura (Euhomoneura) shatalkini

Taxon classificationAnimaliaDipteraLauxaniidae

Papp, 1984

Figures 1–7 , 8–12

Homoneura shatalkini Papp, 1984a: 167. Type locality: Japan. Shatalkin, 2000: 29.

#### Specimens examined.

**CHINA: Ningxia Province** (IMAU): 1♂, Liupan Mountain, 7–8.vii.2008, Jingxian Liu. **CHINA: Jilin Province** (IMAU): 2♂♂, 2♀♀, Changbai Mountain, South slope, 1520–1720 m, 1.viii.2004, Xingyue Liu. **CHINA: Shaanxi Province** (IMAU): 1♂, Zhouzhi, Houzhenzi, 1235 m, 11.viii.2013, Wencheng Chang; 1♂, Zhouzhi, Laoxiancheng, 1846 m, 19.viii.2014, Xiumei Lu; 2♂♂, 2♀♀, Zhouzhi, Laoxiancheng, 1808 m, 12.viii.2013, Wencheng Chang (2 males, 2 females: IMAU); 1♂, Feng County, Huangniupu, light trap, 1501 m, 21.viii.2013, Yuqiang Xi; 1♂, Zhouzhi, Houzhenzi, 1235 m, 11.viii.2013, Xuankun Li; 2♂♂, Zhouzhi, Taibai Mountains, 1648 m, 17.viii.2014, Xunkun Li.

#### Distribution.

New record to China (Ningxia, Jilin, Shaanxi); Japan, Russia.

**Figures 1–7.**
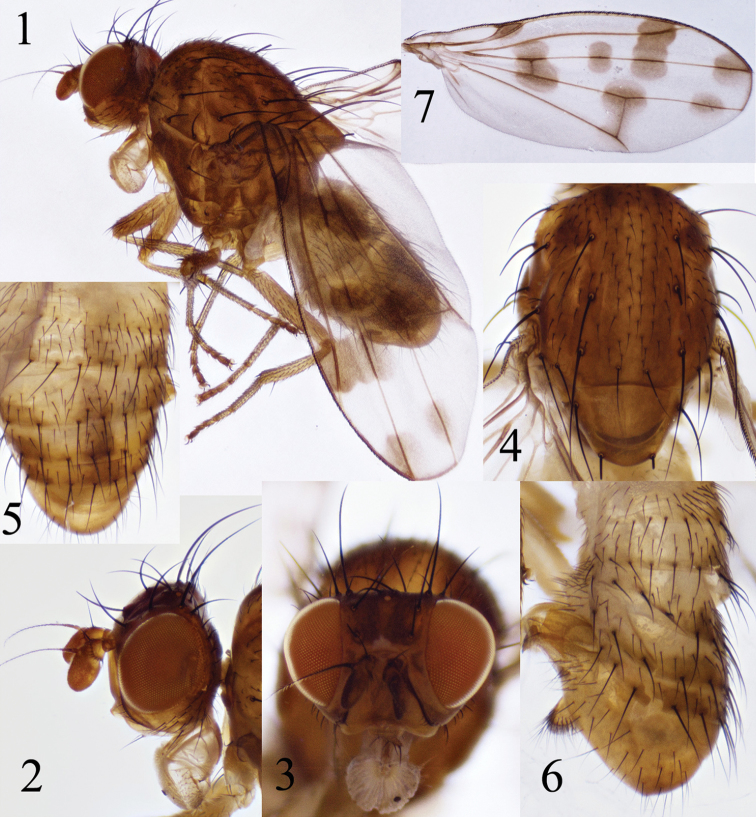
Homoneura (Euhomoneura) shatalkini Papp, 1984. Male from Shaanxi. **1** Habitus, lateral view **2, 3** Head, laeral and anterior view **4** Thorax, dorsal view **5, 6** Abdomen, dorsal and lateral view **7** Wing.

**Figures 8–12.**
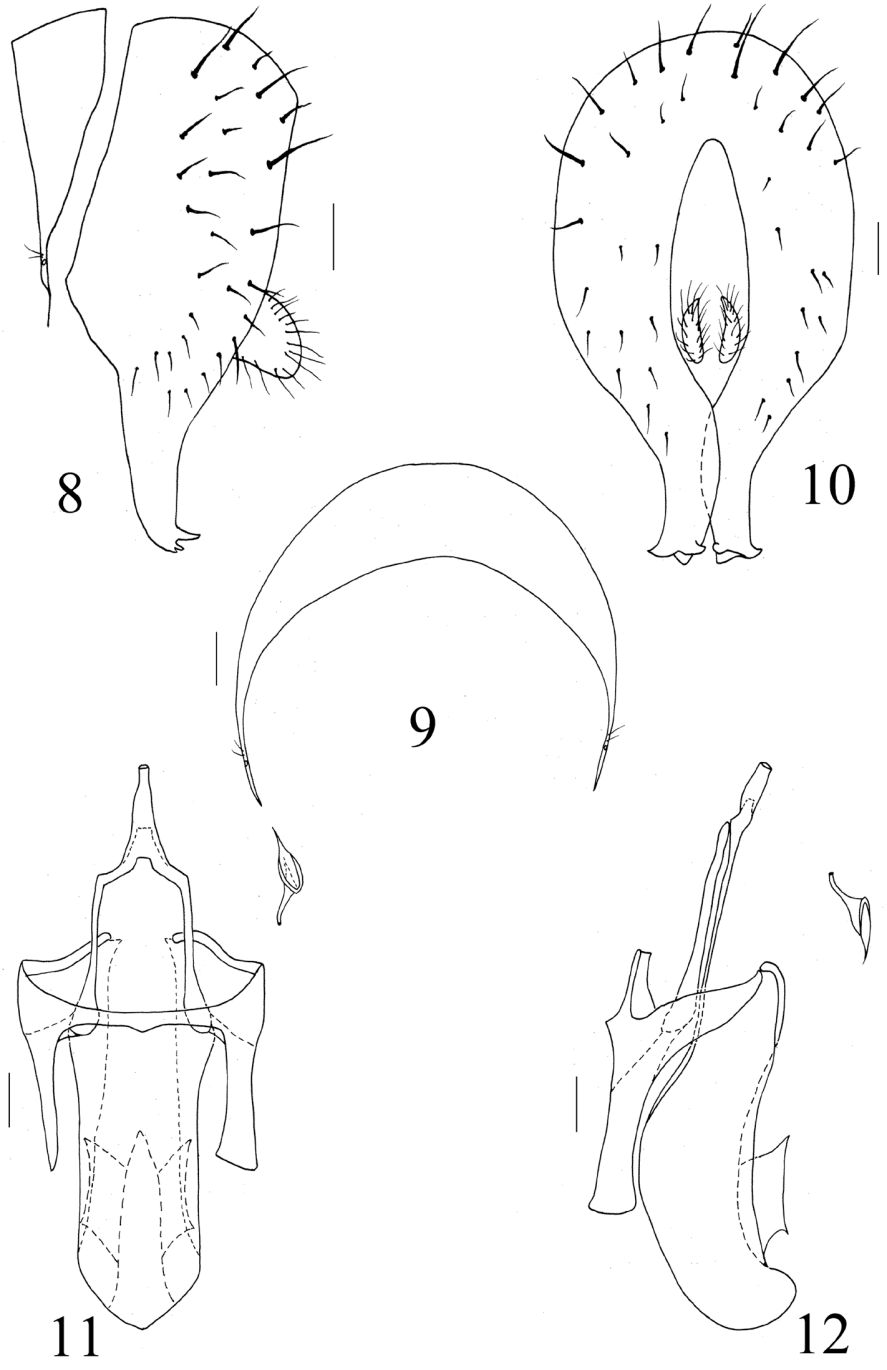
Homoneura (Euhomoneura) shatalkini Papp, 1984. Male from Shaanxi. Male genitalia. **8** Syntergosternite and epandrial complex, lateral view **9** Syntergosternite, anterior view **10** Epandrium, posterior view **11** Aedeagal complex, ventral view **12** Aedeagal complex, lateral view. Scale bar: 0.1 mm.

### Homoneura (Euhomoneura) yanqingensis sp. n.

Taxon classificationAnimaliaDipteraLauxaniidae

http://zoobank.org/EB333015-A744-430C-8448-B06F98ACBB89

Figures 13–19 , 20–24

#### Type material.

Holotype ♂, **CHINA: Beijing** (CAUC): Yanqing County, Songshan, 8.ix.2009, Xiaoyan Liu. Paratypes: **CHINA: Beijing** (CAUC): 7♂♂, 8♀♀, data same as holotype. **CHINA: Shaanxi Province** (IMAU): 1♂, Baoji City, Feng County, Huangniupu, 1501 m, 21.viii.2013, Yuqiang Xi; 1♂, 1♀, Zhouzhi, Houzhenzi, 1235 m, 11.viii.2013, Wencheng Chang; 1♀, Zhouzhi, Laoxiancheng, 1916 m, 19.viii.2014, Xiumei Lu.

#### Diagnosis.

Body yellow. Arista pubescent. Palpus with brown apex. Mesonotum with four strong and long acrostichal setae (two before suture, two after suture). All femora each with a brown irregular apicoventral spot and all tasomeres 3–5 brown. Wing hyaline, with five brown isolated spots.

#### Description.

Male. Body length 3.8–4.2 mm. Wing length 3.7–4.3 mm. Female. Body length 3.8–4.2 mm.Wing length 3.7–4.3 mm.

*Head* pale yellow. Frons with sparse grayish white pruinosity, longer than wide and parallel-sided; ocellar triangle brown, ocellar setae well developed, longer than anterior fronto-orbital setae, anterior fronto-orbital setae shorter than length of posterior one; gena about 1/6 height of eye; antenna yellow; 1^st^ flagellomere yellowish brown, 1.5 times longer than high; arista dark brown except for pale brownish base, ray pubescent, with longest ray as long as 1/3 height of 1^st^ flagellomere. Proboscis yellow, palpus yellow except for brown apex.

*Thorax* yellow. Mesonotum with 1+2 dorsocentral setae; acrostichal setae in irregular six rows, with four strong and long acrostichal setae (two before suture, two after suture); a pair of prescutellar setae shorter than 1^st^ post-sutural dorsocentral setae. Leg mostly yellow, all femora each with a brown irregular apicoventral spot and all tasomeres 3–5 brown. Fore femur with 6–7 posterodorsal setae, 4–5 posterovental seate, and ctenidium with 11–12 short setae; fore tibia with one strong preapical anterodorsal seta and one short apicoventral seta. Mid femur with five anterior setae and one short apical posterior seta; mid tibia with one strong preapical anterodorsal seta and one short apicoventral seta. Hind femur with one preapical anterodorsal seta and three anteroventral setae; hind tibia with one preapical anterodorsal seta and one short apicoventral seta. Wing hyaline, with five brown isolated spots: preapical spot on R_2+3_, apical spot on R_4+5_, subapical spot on M_1_, a cloud on crossvein r-m, and a narrow stripe-like spot on crossvein dm-cu (anterior margin and posterior margin darker than central area); pale brown along the radial sector; subcostal cell pale brown; costa with 2^nd^, 3^rd^ and 4^th^ sections in proportion of 4.5:1.8:1; r-m beyond middle of discal cell; ultimate and penultimate sections of M_1_ in proportion of 1:1.6; ultimate section of CuA_1_ about 1/6 of penultimate. Halter yellow.

*Abdomen* pale yellow, tergites 2–5 (female 2–6) with narrow brown posterior margin, and tergite 3–5 (female 3–6) brownish median spot or absent. Male genitalia: syntergosternite circular with a pair of ventral processes; epandrium broad with dense apical setae, surstylus narrow but slightly broaden at apex; hypandrium slender U-shaped; postgonite long cylindrical; aedeagus broad at middle and bluntly rounded in lateral view, aedeagal apodeme shorter than length of aedeagus.

**Figures 13–19.**
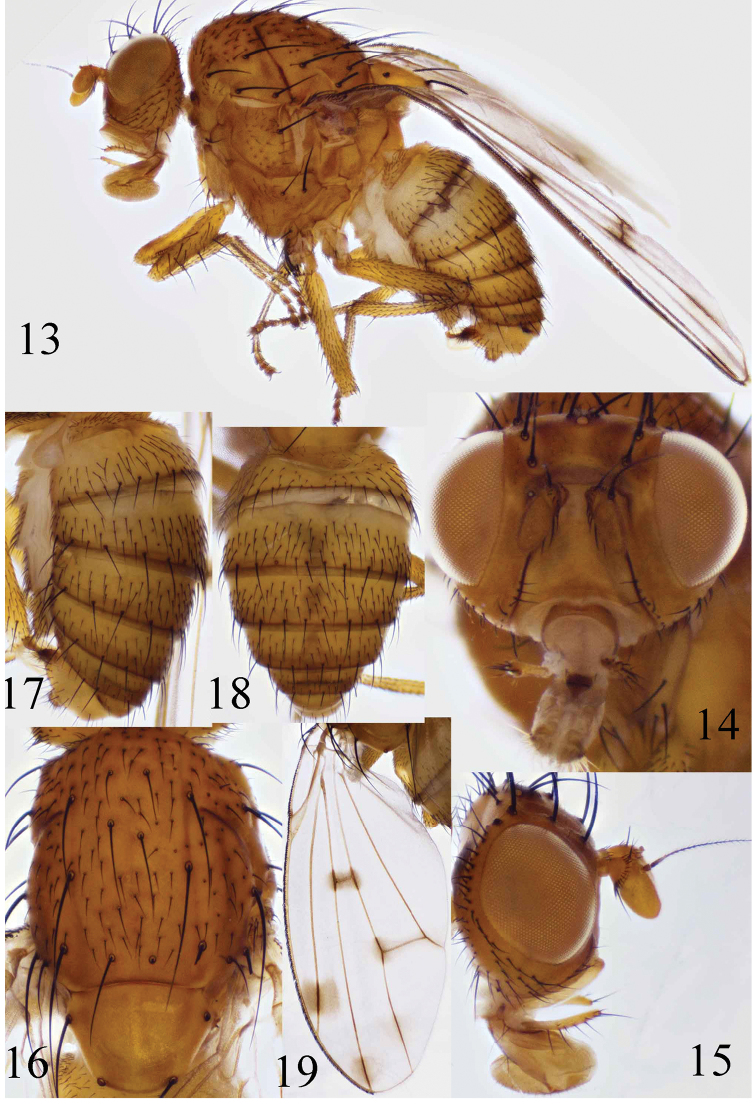
Homoneura (Euhomoneura) yanqingensis sp. n. Paratype male from Shaanxi.**13** Habitus, lateral view **14, 15** Head, anterior and lateral view **16** Thorax, dorsal view **17, 18** Abdomen, dorsal and dorsal view **19** Wing.

**Figure 20–24.**
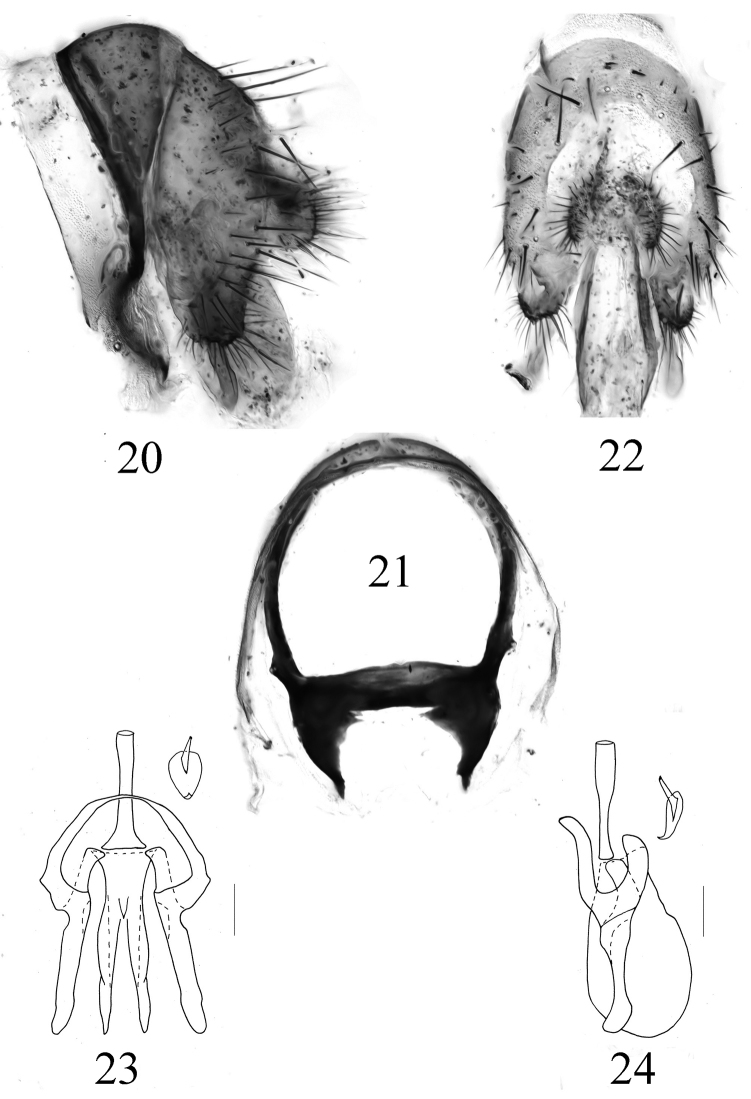
Homoneura (Euhomoneura) yanqingensis sp. n. Paratype male from Shaanxi. Male genitalia. **20** Syntergosternite and epandrial complex, lateral view **21** Syntergosternite, anterior view **22** Epandrium, posterior view **23** Aedeagal complex, ventral view **24** Aedeagal complex, lateral view. Scale bar: 0.1 mm.

#### Etymology.

The new species is named after collection locality.

#### Distribution.

China (Beijing, Shaanxi).

#### Comments.

The new species is similar to Homoneura (Euhomoneura) balluca Sasakawa, 1992 from Malaysia. It can be separated from the latter by the mesonotum having brown stripes, the coxa and femora of legs being brown and the tibiae each having brown rings on both apex, and the abdominal tergite 6 having a pair of brown lateral spots.

### A key to the five known species of the subgenus Euhomoneura in China

**Table:** 

1	Frons black with yellowish grey pruinosity; gena reddish yellow with black spots; male abdominal tergites greyish black without spots, but female abdominal tergites with a pair of reddish brown spots on anterolateral corners	**H. (E.) variipennis Czerny**
–	Frons yellow; gena yellow; abdomen yellow with pale brown spots or absent	**2**
2	Wing with two brown spots on R_4+5_ between vertical level of *r-m* and apical spot	**3**
–	Wing without brown spots on R_4+5_ between vertical level of *r-m* and apical spot	**4**
3	Arista short plumose; male genitalia: surstylus with a sharp and a blunt apical protuberance in lateral view, aedeagus without rectangular dorsal sclerites (see Gao et al. 2003: 194)	**H. (E.) minuscula Gao, Yang & Gaimari**
–	Arista pubescent; male genitalia: surstylus with three sharp processes, aedeagus with a pair of nearly rectangular dorsal sclerites in lateral view (see Papp, 1984: 167).	**H. (E.) shatalkini Papp**
4	Mesonotum with acrostichal setae in irregular six rows, especially four strong and long acrostichal setae (two before suture, two after suture); male genitalia: surstylus blunt without tip and aedeagus separated at apex in posterior view	**H. (E.) yanqingensis sp. n.**
–	Mesonotum without strong central acrostichal setae; male genitalia: surstylus with tip projecting and upturned and aedeagus crossed at apex in posterior view (see Gao et al. 2003: 195)	**H. (E.) xiaolongmenensis Gao, Yang & Gaimari**

## Supplementary Material

XML Treatment for Homoneura (Euhomoneura) shatalkini

XML Treatment for Homoneura (Euhomoneura) yanqingensis
